# QA of intensity-modulated beams using dynamic MLC log files

**DOI:** 10.4103/0971-6203.25668

**Published:** 2006

**Authors:** M. Dinesh Kumar, N. Thirumavalavan, D. Venugopal Krishna, M. Babaiah

**Affiliations:** Department of Radiation Oncology, Yashoda Cancer Institute, Secunderabad, Andhra Pradesh, India

**Keywords:** DMLC field files, dynalog files, gamma index, IMRT

## Abstract

To evaluate the utility of Dynalog file information for planar dose verification in IMRT QA, a program is developed to convert Dynalog file data to DMLC field files. For this study, five predefined fluencies are planned and delivered using Varian, Eclipse 3D planning system and 6MV photon beam of Varian, Clinac DMX linear accelerator. To measure planar dose distribution, Kodak, EDR2 films are exposed in similar setup as planning setup. Dynalog files are recorded for each delivery and converted into DMLC field files using in-house program. Delivered dose distributions are calculated using DMLC field files from Dynalog files. Planned, Measured and Delivered dose distributions are compared using gamma evaluation in Scanditronix, Omni Pro IMRT software. The Planned and Delivered planar dose distributions agree within 2% dose difference and 2 mm DTA. Measured dose distributions agree within 4% dose difference and 4 mm DTA with Planned dose distribution. Our results show Dynalog file as a promising tool for dynamic IMRT QA.

## Introduction

A recent advance in external beam radiation therapy is the use of non-uniform intensity photon fields to produce dose distribution conformed to complex targets. One of the IMRT delivery methods is sliding window technique using dynamic multi-leaf collimator, where leaves are in motion while the beam is ON.

The basic steps in Intensity-Modulated Radiotherapy (IMRT) are target and OAR delineation, generation of intensity-modulated fields based on given dose volume constraints, 3D dose calculations and delivery of intensity-modulated beams. All these steps require strict commissioning and periodic quality assurance. The detailed IMRT QA is described by LoSasso *et al.*[1] QA of delivery technique is broadly divided into machine and plan-specific (patient-specific). The detailed dosimetric comparison between planned and measured planar dose distribution is the core of IMRT QA for planning and delivery system. The lengthy and tiresome procedure in plan-specific IMRT QA is planar dose verification. Conventionally, we do this with Film dosimetry. To reduce the taxing without hampering the quality of this QA procedure, many automation methods are evaluated using 2D Detectors arrays, Gel dosimeter and Electronic Portal imaging device (EPID). In this way, utility of the Dynalog files for routine IMRT QA was studied by Litzenberg, *et al.*[2] Li, *et al.*[3] validated the Dynamic MLC log files for IMRT QA using a two-dimensional diode array. Here we report automation of planar dose verification using Dynamic MLC log files created by MLC controller after each IMRT delivery.

## Materials and Methods

In our institute, we do IMRT planning and delivery by using Eclipse 3D planning system with Helios Inverse planning module and photon beams 6MV and 16MV from Varian, Clinac DMX linear accelerator with Millennium 52 leaves MLC.

### The Dynamic Multi-Leaf Collimator (DMLC)

The computer-controlled single-focused collimating device consists of two opposing banks of 26 pairs of rounded-end tungsten leaves of 1 cm width at isocenter. This collimator is mounted on CLINAC DMX linear accelerator as a tertiary collimator. A dynamic treatment with DMLC is a treatment technique where both the dose rate and the speed of the leaves are continually adjusted by the MLC control system during the Beam ON. The dynamic treatment information is given to the DMLC control system through ASCII text file. These text files are called DMLC field files and they contain the sequence of MLC shapes that are to be delivered per field. DMLC field file contains group of MLC pattern fields with header information of field number and dose index. The dose index is the fraction of the MU to be delivered using particular MLC shape and it will be 0 for the first field and 1 for the last field. Since DMLC shape is constantly changing, each instantaneous MLC shape is determined by linear interpolation between the field shapes. Further information is available in Varian User Manual, DMLC implementation guide.[4]

For 06 MV photon beam, DMLC's Dosimetric leaf gap and Dynamic leaf tolerance values are 1.6 mm and 2 mm respectively. MLC readout calibration is within 1 mm accuracy.

### Dynamic log files

**Figure 1:**
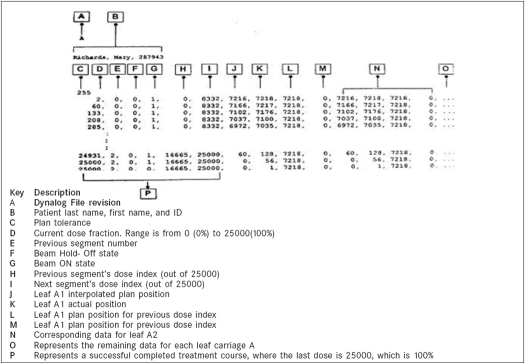
Dose dynamic treatment dynalog file contents

A Dynamic MLC log file (Dynalog file) is a record of DMLC delivery details recorded at every 0.05 s by the DMLC controller for a dynamic treatment. The controller assigns unique file name to the generated Dynalog files and separate files are created for A and B MLC banks. Figure 1 shows a typical Dynalog record, where header line contains patient information and file revision information. Every line starts with dose index fraction at the time of registration and contains information of

i) Beam ON/OFF states, ii) previous and next field index values, iii) leaf actual position and iv) leaf position at previous and next index values.

In this record, the leaf position values are mentioned in units of ‘mm X100’ at the leaf plane and dose index is 0 and 25000 for dose index 0 and 1 respectively. A complete file description can be found at Dynalog File Viewer reference guide, Varian (2003).[5]

### Eclipse IMRT planning process

IMRT Planning in Varian, Eclipse 3D planning system is a three-fold process: i) Helios inverse planning module is used to generate optimal fluence (desired) patterns for each beam on the basis of defined dose volume constraints. ii) The Leaf Motion Calculator (LMC) converts these optimal fluence patterns into actual fluence (deliverable) patterns and DMLC field files by considering the physical and dosimetric constraints of DMLC. iii) Forward planning software uses these actual fluence patterns for dose calculation.

### Dynalog-to-DMLC field file converter (DMC)

**Figure 2:**
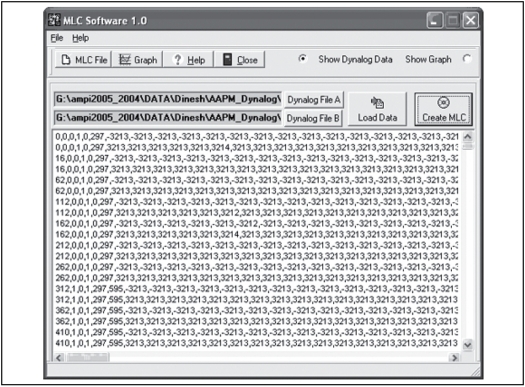
The operating window of dynalog to DMLC field file converter

We have written software to convert the information of actual leaf position and fractional dose index of each record into a MLC shape of DMLC field files. This software is written in Delphi programming language, which is similar to ‘Visual Basic.’ While programming, we have taken into account physical and dosimetric aspects of DMLC. Figure 2 shows the operating window of Dynalog-to-DMLC field file converter. We can run the program with the input of Dynalog files of A and B bank MLC and the header information of the field, whereas to create one DMLC field (MLC shape), leaf actual position information with same dose index fraction in A and B MLC bank Dynalog file records are taken. The maximum number of MLC shapes allowed per DMLC field file is 320 and in a Dynalog file, there will be more than 320 records (as they are recorded at intervals of 0.05 s). By considering above constraints, it is programmed to create a group of DMLC field files per Dynalog file and each DMLC field file is weighed with their contribution to total dose. It can also create an index file with the information of DMLC field file weights and its sequence. This software has the capability to visualize the DMLC field file's motion. DMC program allows us to define DMLC tolerance of DMLC field files.

### Dynalog File Viewer (DFV)

Dynalog File Viewer (DFV) is a utility program that presents the data from a Dynalog file in a graphical format. A detailed description of the software can be found at Dynalog File Viewer reference guide, Varian.[5]

### Gamma evaluation

It is very difficult to compare the IMRT planar dose distributions by dose difference in low-gradient region and DTA (distance to agreement) in high gradient region independently due to unsystematic presence of low and high gradient regions. The gamma method presented,[6,7] by considering the complementary sensitivity of dose difference and DTA in low and high gradient regions respectively, is useful for the evaluation of IMRT planar dose distribution.

**Figure 3:**
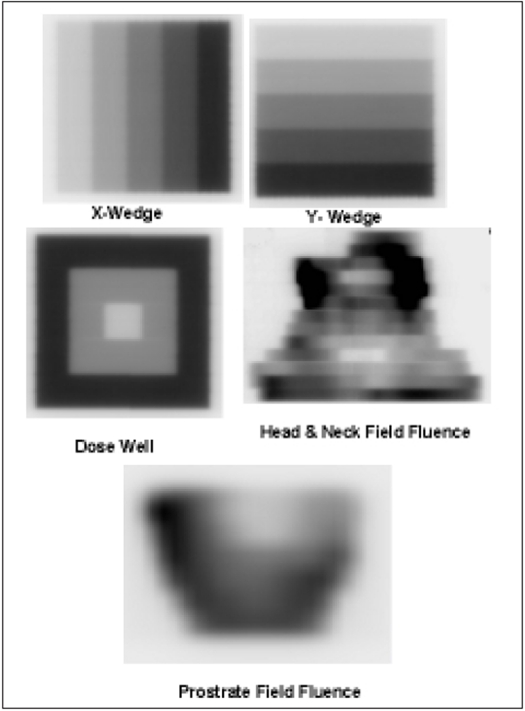
Fluencies of test patterns and clinical IMRT plan fields used in this study

For this study, test patterns to produce the dose distribution like X wedge (5 intensity levels of 2 cm width in leaf motion direction), Y wedge (5 intensity levels of 2 cm width in perpendicular to leaf motion direction) and Dose well (low intensity region in center surrounded by high intensity regions) over 10 × 10 cm^2^ are created in an Excel Spread sheet. Two optimal fluencies from Prostate and Head and Neck IMRT plans are taken. These five fluencies are showed in the Figure 3. All these fluencies are imported into independent IMRT plans generated for each fluence in Eclipse 3D planning system. For all planning fields, isocenter is placed at 5 cm from the phantom surface (i.e, SSD is 95 cm) and plans are normalized to a reference point in isocenter plane. Beam module of Clinac DMX, 06MV photon beam and Millennium 52 DMLC is used for dose calculation. All plans are moved to Varian, Clinac DMX 4D treatment unit for execution.

**Figure 4:**
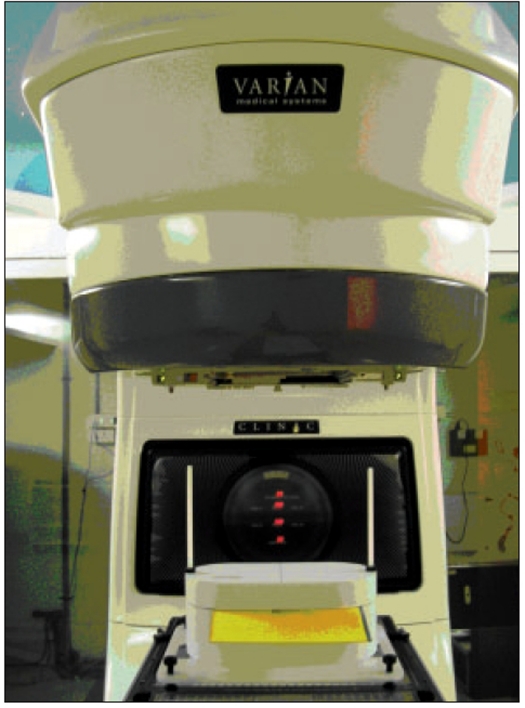
Planar dose distribution measurement setup to expose Kodak EDR2 films. Where the film to Source distance is 100 cm and Phantom surface to Film distance is 5 cm

Delivery setups for all IMRT plans are analogous to planning setups and to obtain Measured planar dose distribution, Kodak EDR2 films are exposed in a solid phantom as shown in Figure 4, where the Film-to-Source distance is 100 cm and Phantom Surface to Film distance is 5 cm. For each delivered field, Dynamic log files are recorded and these log files are converted into DMLC field files by DMC (Dynalog-to-DMLC field file converter). All DMLC field files are imported into Eclipse 3D planning system and Delivered dose distributions are calculated in similar manner as Planned dose distributions.

Dose distributions generated using fluence patterns (Planned dose distribution) and DMLC field files from Dynalog files (Delivered dose distribution) are compared for each pattern in Eclipse Plan evaluation module.

Exposed EDR2 films are processed and analyzed using Vidar scanner and Scanditronix, Omni pro IMRT software to obtain Measured dose distributions. Planned, Delivered and Measured dose distributions are analyzed by gamma evaluation method in Scanditronix, Omni pro IMRT software.

Dynamic log files of each fluence pattern are analyzed using Dynalog file Viewer program and MLC positional Error Histograms and RMS values are recorded.

To understand DMLC delivery, as part of Daily QA, uniform field is delivered dynamically with 5 mm sweeping gap and recorded Dynalog files are analyzed with DFV program. Percentage of errors within the leaf position error of 1 mm, maximum error RMS and leaf having leaf-positional errors more than 2 mm are recorded and compared with base-line values.

## Results and Discussion

Planned, Measured and Delivered Dose distributions are compared using gamma evaluation for X wedge, Y Wedge, Dose well. Prostate and Head and Neck fluencies are shown Figures 5–9 respectively.

**Figure 5:**
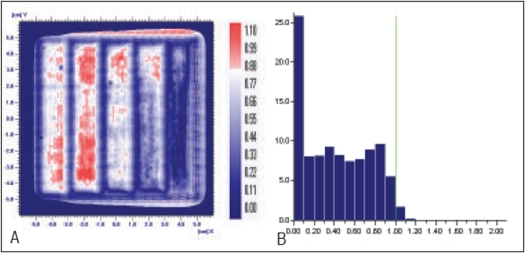
A) Comparison of X wedge's planned and measured dose distribution using A) gamma distribution and B) gamma histogram with criteria of 3% and 3mm dose difference and DTA respectively.

**Figure 5:**
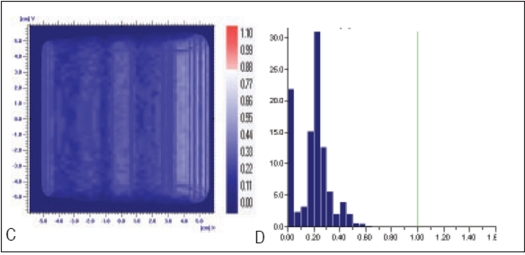
Comparison of X wedge's planned and delivered dose distribution using C) gamma distribution and D) gamma histogram with criteria of 1% and 1mm dose difference and DTA respectively

**Figure 6:**
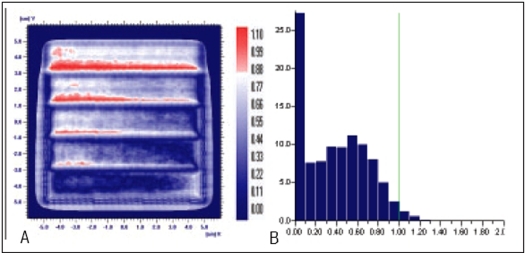
Comparison of Y wedge's planned and measured dose distribution using A) gamma distribution and B) gamma histogram with criteria of 3% and 3mm dose difference and DTA respectively

**Figure 6:**
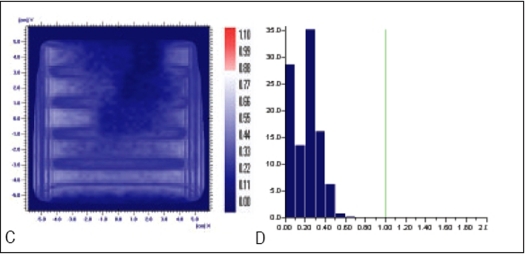
Comparison of Y wedge's planned and delivered dose distribution using C) gamma distribution and D) gamma histogram with criteria of 1% and 1mm dose difference and DTA respectively

**Figure 7:**
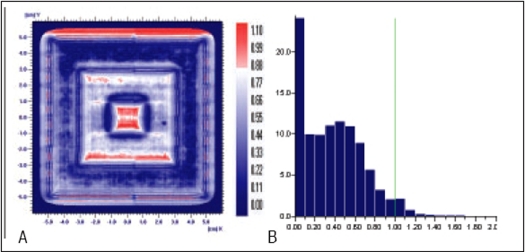
Comparison of Dose well's planned and measured dose distribution using A) gamma distribution and B) gamma histogram with the criteria of 3% and 3mm dose difference and DTA respectively

**Figure 7:**
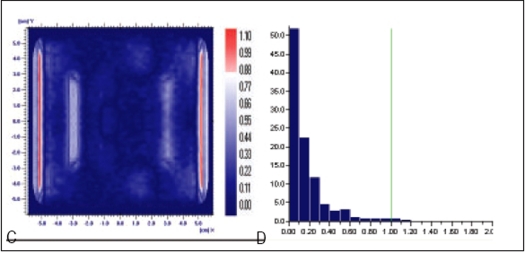
Comparison of Dose Well's planned and delivered dose distribution using C) gamma distribution and D) gamma histogram with the criteria of 1% and 1mm dose difference and DTA respectively

**Figure 8:**
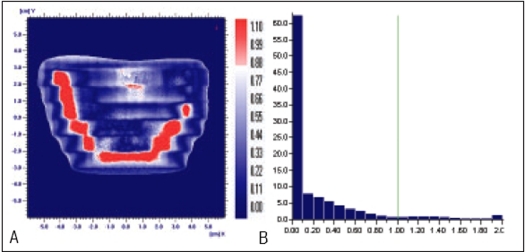
Comparison of prostate field's planned and measured dose distribution using A) gamma distribution and B) gamma histogram with criteria of 4% and 4mm dose difference and DTA respectively

**Figure 8:**
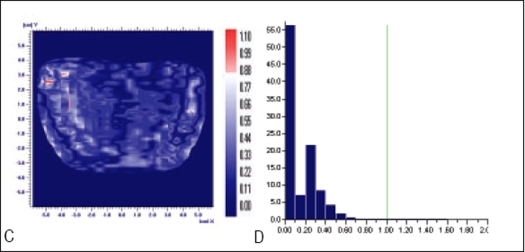
Comparison of prostate field's planned and delivered dose distribution using C) gamma distribution and D) gamma histogram with criteria of 2% and 2mm dose difference and DTA respectively

**Figure 9:**
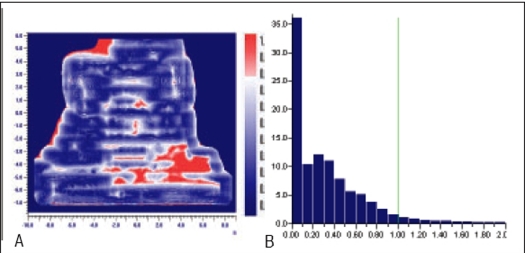
Comparison of H&N field's planned and measured dose distribution using A) gamma distribution and B) gamma histogram with criteria of 4% and 4mm dose difference and DTA respectively

**Figure 9:**
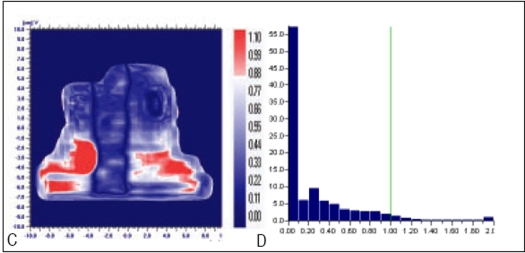
Comparison of Head & Neck field's planned and delivered dose distribution using C) gamma distribution and D) gamma histogram with criteria of 2% and 2mm dose difference and DTA respectively

In Figures 5–9, Sections A and B show the difference between Planned and Measured dose distributions in gamma distribution and gamma histogram. Sections C and D show the difference between Planned and Delivered dose distributions in gamma distribution and gamma histogram.

For X wedge, Y wedge and Dose well fluencies, Planned and Measured dose distributions agree within the criteria of 3% dose difference and 3 mm DTA and Delivered dose distributions agree within the criteria of 1% dose difference and 1 mm DTA with Planned distributions.

For prostate and Head and Neck fluencies, Planned and Measured dose distributions agree within the criteria of 4% dose difference and 4 mm DTA and Delivered dose distributions agree within the criteria of 2% dose difference and 2 mm DTA with Planned distributions. Correlation between two distributions for a given criteria is acceptable only if more than 95% data points in the distribution have gamma index (γ) ≤ 1.

Table 1 shows the details of gamma evaluation and leaf-positional error for all fluencies. The inference of Table 1 is that dose differences between Planned and Delivered dose distributions are in correlation with Error RMS and Error Histogram.

**Table 1:** All fluence pattern's details of gamma evaluation and leaf positional errors

*Fluence*	*Gamma evaluation*							
	
	*Planned vs Delivered criteria*			*Planned vs measured*			*Leaf positional errors*	
			
	*% Dose difference*	*DTA mm*	*% of Data points gamma <1*	*% Dose difference*	*DTA mm*	*% of Data points gamma <1*	*% of counts Having error less than 1 mm*	*Maximum error RMS*
X wedge	1	1	100	3	3	97	97	0.047
Y wedge	1	1	100	3	3	98	96	0.056
Dose well	1	1	99	3	3	96	98	0.032
H&N	2	2	96	4	4	96	98	0.041
Prostate	2	2	96	4	4	95	99	0.034

In daily QA, analyses of sweeping field's Dynalog files show more than 95% positional errors are within 1 mm, maximum Error RMS values are within 0.04 and there is no Beam Hold Offs while on treatment.

Dosimetric differences between Planned and Measured dose distribution can be attributed to three sources; i) dose calculation errors in the treatment planning system, ii) error caused by dosimeter used and iii) errors in the delivery system. Errors due to first two sources can be studied independently with other methods. Basic aim of planar dose distribution comparison is to study delivery errors. Therefore, this method is very useful to study the delivery errors of dynamic treatments within the uncertainties due to MLC calibration.

With this method, we can study the delivery accuracy of daily IMRT treatment. Further automation of this program to calculate dose distribution can become an independent IMRT QA tool. For Pretreatment QA, Dynalog files for each IM field can be obtained in dry run. To study day-to-day IMRT delivery accuracy, Dynalog files created at the time of IMRT treatment will be used so that the additional time spent by the physics staff for plan-specific QA can be reduced.

The Dynalog files contain significant information of the delivered dynamic treatment. This can be used to understand delivery errors and accuracy of dynamic treatment. The validity of Dynalog file data is dependent on the MLC readout calibration. By employing routine QA of MLC calibration and independent checks for IMRT planning, information in Dynalog files can be effectively used for IMRT QA automation.

## Conclusion

We studied a method to evaluate Dynalog files as IMRT QA tool and our result shows that the information in Dynalog files can be used for IMRT routine and plan-specific QA. Automation of IMRT QA procedure using Dynalog files reduces the time spent for IMRT QA by physics staff and provides a tool to analyze entire IMRT day-to-day delivery. Within the uncertainties of MLC calibration, Dynalog file is a promising tool for IMRT QA automation.
